# Clinical and radiographic predictors of acute compartment syndrome in the treatment of tibial plateau fractures: a retrospective cohort study

**DOI:** 10.1186/s12891-017-1680-4

**Published:** 2017-07-18

**Authors:** Axel Gamulin, Anne Lübbeke, Patrick Belinga, Pierre Hoffmeyer, Thomas V. Perneger, Matthieu Zingg, Gregory Cunningham

**Affiliations:** 1 Department of Surgery, Division of Orthopaedic and Trauma Surgery, University Hospitals of Geneva, 4 Rue Gabrielle-Perret-Gentil, CH-1211 Geneva 14, Switzerland; 2 Division of Clinical Epidemiology, University Hospitals of Geneva, 4 Rue Gabrielle-Perret-Gentil, CH-1211 Geneva 14, Switzerland

**Keywords:** Tibial plateau fracture, Proximal tibia fracture, Acute compartment syndrome, Fasciotomy, Risk factors

## Abstract

**Background:**

The aim of the study was to evaluate the relation between demographic, injury-related, clinical and radiological factors of patients with tibial plateau fractures and the development of acute compartment syndrome.

**Methods:**

All consecutive adult patients with intra-articular tibial plateau fractures admitted in our urban academic medical centre between January 2005 and December 2009 were included in this retrospective cohort study. The main outcome measurement was the development of acute compartment syndrome.

**Results:**

The charts of 265 patients (mean age 48.6 years) sustaining 269 intra-articular tibial plateau fractures were retrospectively reviewed. Acute compartment syndrome occurred in 28 fractures (10.4%). Four patients presented bilateral tibial plateau fractures; of them, 2 had unilateral, but none had bilateral acute compartment syndrome. Non-contiguous tibia fracture or knee dislocation and higher AO/OTA classification (type 41-C) were statistically significantly associated with the development of acute compartment syndrome in multivariable regression analysis, while younger age (<45 years), male sex, higher Schatzker grade (IV-V-VI), higher tibial widening ratio (≥1.05) and higher femoral displacement ratio (≥0.08) were significantly associated in the analysis adjusted for age and sex.

**Conclusions:**

Two parameters related to the occurrence of ACS in tibial plateau fractures were highlighted in this study: the presence of a non-contiguous tibia fracture or knee dislocation, and higher AO/OTA classification. They may be especially useful when clinical findings are difficult to assess (doubtful clinical signs, obtunded, sedated or intubated patients), and should rise the suspicion level of the treating surgeon. In these cases, regular clinical examinations and/or intra-compartmental pressure measurements should be performed before and after surgery, even if acute compartment syndrome seemed unlikely during initial assessment. However, larger studies are mandatory to confirm and refine both factors in predicting the occurrence of acute compartment syndrome.

## Background

Tibial plateau fractures account for approximately 1% of all fractures [1]. Schatzker and AO/OTA classifications are widely used for their description [2, 3]. Tibial plateau fractures represent a broad range of injury pattern, from low-energy mainly undisplaced, isolated lateral plateau fractures, to high-energy displaced, medial plateau, bicondylar or epiphyso-metaphyso-diaphyseal fractures [4–6]. The amount of energy released in the bone and soft-tissues during trauma may determine associated soft-tissue lesions such as skin lacerations (open fracture) and acute compartment syndrome (ACS) [4–7]. Occurrence of ACS may be as high as 12% in overall tibial plateau fractures [8–11], and as high as 53% in higher-energy fracture subgroups (Schatzker IV, V and VI) [8, 11–15].

Trauma patients, especially those involved in high-energy polytrauma, may be intubated and/or sedated and/or obtunded at time of assessment by the orthopaedic surgeon. In such conditions, usual valuable clinical signs for diagnosing ACS (pain out of proportion with the findings on physical examination and exacerbated by passive stretch of the muscles in the area of the injury) may be impossible to evaluate [16, 17]. In addition to intra-compartmental pressure (ICP) measurement, alternative predictors of impending ACS need to be determined.

The literature regarding risk factors for the occurrence of ACS after tibial plateau fractures is scarce. Radiographic determinants have been recently reported, such as higher Schatzker and AO/OTA grades, increased tibial widening ratio (TWR), increased femoral displacement ratio (FDR), increased fracture length and associated fibular fracture [8, 11–15]. Identified risk factors for ACS following tibial shaft fractures include young age, male sex, mechanism of injury, high-energy trauma and open fracture [9, 16–19]. However, the presence of a concomitant non-contiguous tibia fracture or knee dislocation may be an additional valuable indicator for high-energy trauma associated with extended musculoskeletal damage, and may hence also be a predictive factor for development of ACS. To the authors’ knowledge, no study has to date evaluated these potential risk factors simultaneously.

The objective of this study was therefore to evaluate the relation between key demographic, injury-related, clinical and radiographic factors in patients with tibial plateau fractures and the subsequent development of ACS.

## Methods

### Ethics statement

Approval from the local institutional research ethics board was obtained prior to initiation of the study.

### Study participants

This retrospective cohort study was conducted in a 1900-bed urban academic medical centre providing primary and tertiary care for 500,000 inhabitants. All patients admitted for a proximal tibia fracture were identified using the institutional hospitalization diagnoses database, which lists all patients diagnoses upon hospitalization. Inclusion criteria were: 1) a proximal tibia fracture as defined by the AO/OTA classification code 41 [1]; 2) trauma as a cause of fracture; 3) presentation to our institution between January 2005 and December 2009; and 4) age > 16 years old at the time of injury. At this point, 319 fractures were eligible. Exclusion criteria were: 1) extra-articular fractures (AO/OTA type 41-A), as these are commonly excluded from studies about tibial plateau fractures (18 fractures); 2) open growth plates at the time of initial presentation (one fracture); 3) presentation to our institution more than 24 h after initial trauma (patient-based late presentation or referral from another institution; 27 fractures); 4) pathological (primary neoplasm, metastasis) or spontaneous fracture (preceding and as cause of the fall) (one fracture); 5) periprosthetic or peri-implant fracture (two fractures); 6) available radiographic workup inadequate for intra-articular involvement evidence and Schatzker and AO/OTA classifications; 7) above or below knee amputation within the first 24 h after the trauma; and 8) death within the first 24 h after the trauma (one fracture). Finally, 265 patients with 269 fractures were included in the analysis.

### Outcome

The outcome was presence of ACS leading to fasciotomy. Standard clinical evaluation was performed on every patient, at admission in the emergency room and during every preoperative and postoperative visit. The attending orthopaedic surgeon established the diagnosis, with or without the use of ICP measurements [20, 21]. ICP monitoring was not used routinely but only in selected patients: those with equivocal clinical signs and those intubated, sedated or obtunded [20, 21]. When indicated, ICP was measured in each of the four leg compartments using an Intra-Compartmental Pressure Monitor (Stryker Osteosynthesis AG, Biberist, Switzerland). Patients with clinical signs of ACS and/or with pathological ICP values (a difference of less than 30 mmHg between diastolic blood pressure and ICP) were taken to the operative room for four compartments fasciotomy, and the presence or absence of muscle bulging at the time of fasciotomy was reported in the operative notes. For the purpose of this study, ACS was defined as pathological ICP values before fasciotomy, and/or by the presence of muscle bulging at the time of fasciotomy. When ICP was not measured (alert and collaborative patients with unequivocal clinical signs), muscle bulging had to be reported in the operative notes to confirm ACS. If the muscle aspect was not described in the operative notes, the diagnosis of ACS was based on ICP measurements. The diagnosis of ACS was not confirmed if fasciotomy was performed without prior ICP measurements, and if there was no muscle bulging at the time of surgery.

### Variables of interest

Patient-related (age, sex) and fracture-related (mechanism of injury, closed vs. open fracture and Gustilo classification [22, 23] in case of open fracture) variables were extracted from the patients’ charts. According to the mechanism of injury, trauma was further classified as either “fall from own height” or “other” (falls from more than one meter high, sports injuries, road traffic accidents, crushes, construction site accidents, farming accidents) in an attempt to differentiate between low and high-energy trauma. For statistical analysis, fractures were considered in two ways: 1) either closed or open; and 2) in three categories (closed, Gustilo type 1 and 2, and Gustilo type 3).

**Fig. 1:**
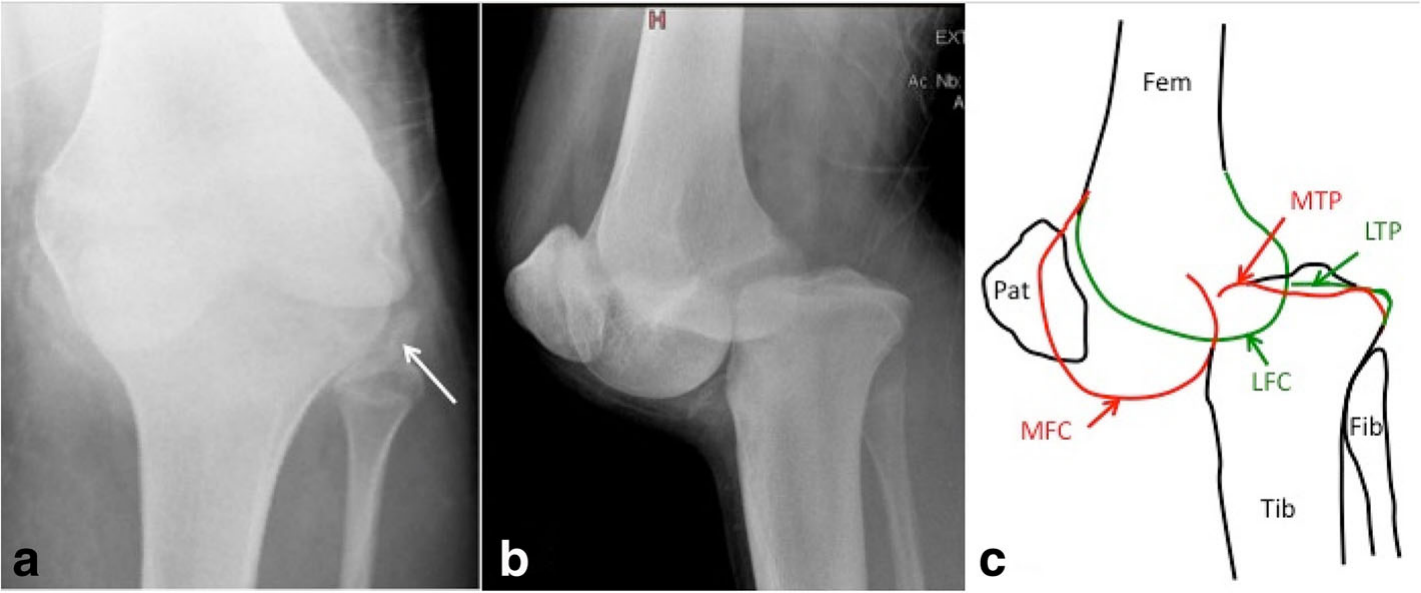
Plain antero-posterior (**a**) and lateral (**b**) radiographs of the left knee of a 17 year old male with a complete dislocation of the knee and a lateral tibial plateau fracture (Schatzker II, AO/OTA 41-B3.1). The white arrow shows the lateral plateau fragment on the antero-posterior view. Schematic diagram (**c**) of the lateral radiograph showing dislocation on both medial (red drawing) and lateral (green drawing) femoro-tibial joint lines. The line representing the lateral tibial plateau is interrupted due to the fracture of the anterior part of the lateral plateau. *Fem*: femur; *Fib*: fibula; *LFC*: lateral femoral condyle; *LTP*: lateral tibial plateau; *MFC*: medial femoral condyle; *MTP*: medial tibial plateau; *Pat*: patella; *Tib*: tibia

**Fig. 2:**
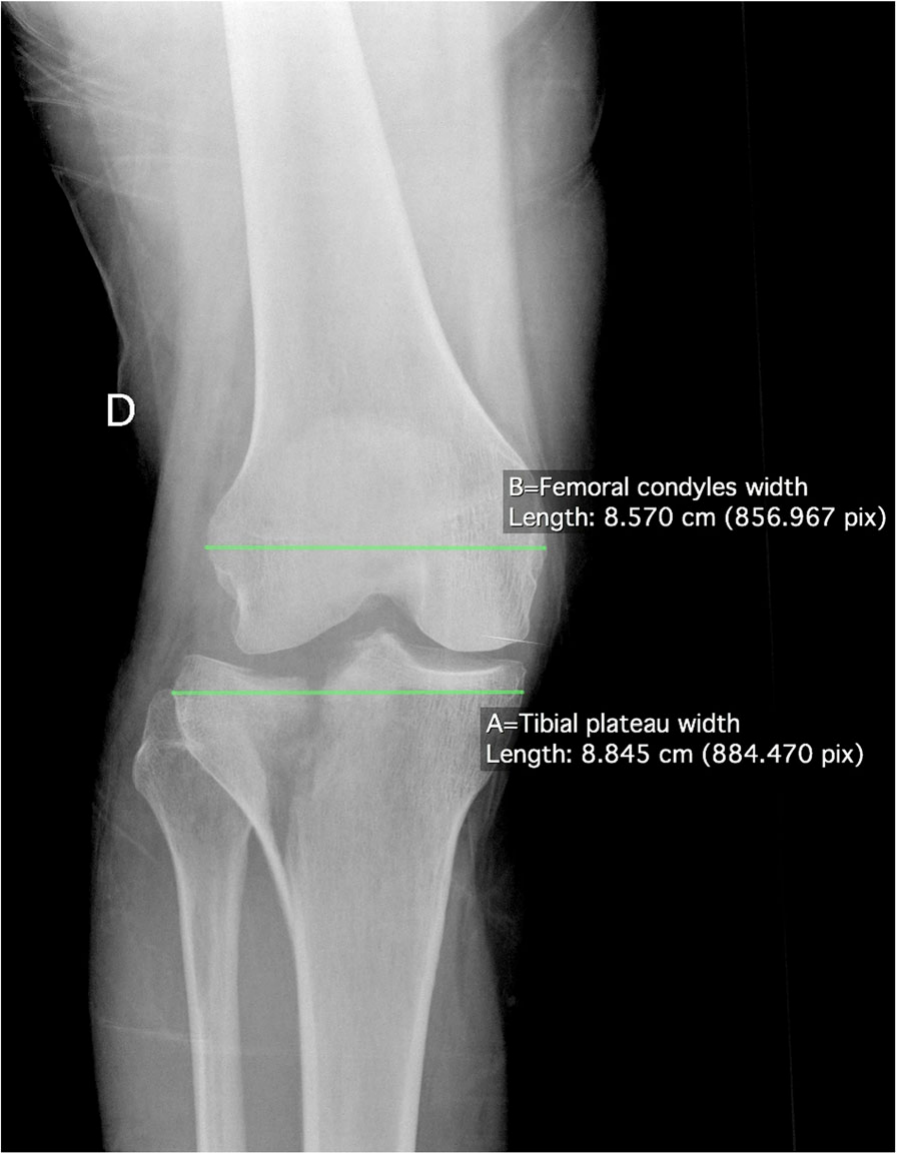
Plain antero-posterior radiograph of a right knee with a Schatzker II, AO/OTA 41-B3.1 fracture. Line A represents the tibial plateau width (the width of the tibial plateau at its widest point). Line B represents the femoral condyles width (the width of the femoral condyles at their widest point). To compensate for any magnification error, a unitless ratio was calculated in order to depict tibial widening: Tibial Widening Ratio (TWR) = Line A/Line B. In the case illustrated, TWR was 8.85/8.57 = 1.03

**Fig. 3:**
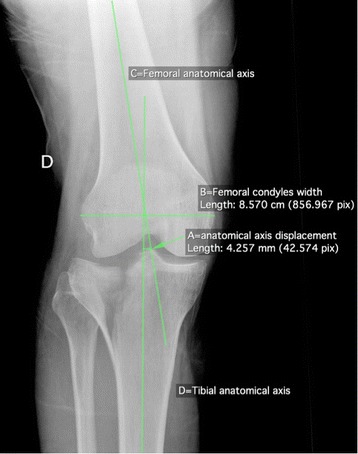
Plain antero-posterior radiograph of a right knee with a Schatzker II, AO/OTA 41-B3.1 fracture. Line A represents the displacement of the tibial anatomical axis (Line D) to the femoral anatomical axis (Line C), measured at the best estimate of the joint line which is 1.5 cm proximal to the fibular head. Line B represents the femoral condyles width (the width of the femoral condyles at their widest point). To compensate for any magnification error, a unitless ratio was calculated in order to depict femoral displacement: Femoral Displacement Ratio (FDR) = Line A/Line B. In the case illustrated, FDR was 0.43/8.57 = 0.05

Based on the initial set of radiographs, Schatzker and AO/OTA classifications were determined, as well as the presence or absence of a non-contiguous tibial shaft or pilon fracture, or knee dislocation. Schatzker classification was divided in two groups: Schatzker I to III (exclusively lateral plateau fractures, involving less resistant bone than the medial plateau which is more sclerotic due to the physiologically slightly varus mechanical axis), and Schatzker IV to VI (involving either the medial plateau or a complete metaphyseal disruption, thus probably representing a higher energy pattern) [4–6]. Only the first level of the AO/OTA classification (41-B vs. 41-C) was used for the statistical analysis. Radiographic analysis of all cases was performed by a single trauma-fellowship trained orthopaedic surgeon, who was blinded to the outcome. Moreover, since intra- and interobserver reliability of Schatzker and AO/OTA classifications has been shown to be poor (moderate to substantial) in a recent study [24], a new reliability assessment of these both classifications was performed: an Internet based random integer generator (http://www.random.com) was used to randomly select 30 cases among the 269 fractures. As cases were picked independently from each other, duplicates may occur that were excluded from the reliability assessment. The trauma-fellowship trained orthopaedic surgeon performed new Schatzker and AO/OTA classifications on the randomly picked radiographs a second time, 6 months after the first analyse (intraobserver reliability). An orthopaedic chief resident also performed the above-mentioned classifications on the selected series (interobserver reliability). Non-contiguous tibial shaft or pilon fractures were defined as a second fracture complex separated from the tibial plateau fracture and located distal to the metaphyso-diaphyseal junction, thus representing a different pattern from Schatzker VI fractures [3]. Knee dislocation was defined as dislocation on both medial and lateral femoro-tibial joint lines (Fig. 1). This injury pattern has not yet been described in the literature to the best of our knowledge and must thus be very rare. It possibly represents a specific injury mechanism, with energy dissipated on one hand in the bone (producing the tibial plateau fracture) and on the other hand in the soft tissue structures supporting the joint (tearing the ligaments and producing a complete knee dislocation). This differs from the more common knee fracture-dislocation described by Moore, where only one (medial or lateral) joint line is dislocated, while the other stays reduced [25]; the tear in the supporting ligament of the dislocated compartment is possibly a direct consequence of displacement of the fracture fragment, without additional energy dissipated in the soft tissue structures supporting the joint. In this sense, tibial plateau fracture associated with complete knee dislocation might be a specific injury pattern comparable to segmental fractures, and we therefore included associated non-contiguous tibia fractures and knee dislocation in the same group. In order to control the radiographic evaluation of complete knee dislocation, both cases showing this condition were inserted in the group of cases randomly picked for reliability assessment of Schatzker and AO/OTA classifications, and were reviewed by the trauma-fellowship trained orthopaedic surgeon and the orthopaedic chief resident. Specific radiographic measurements (Figs. 2 & 3) were taken from the initial antero-posterior plain radiograph of the knee after the limb was grossly aligned, as described by Ziran et al. [11]. The following measurements were obtained using a dedicated open-source PACS workstation DICOM viewer (OsiriX, Pixmeo Sàrl, Bernex, Switzerland): 1) the width of the tibial plateau at its widest point; 2) the width of the femoral condyles at their widest point; 3) TWR (width of the tibial plateau at its widest point/width of the femoral condyles at their widest point); 4) anatomical axis displacement (absolute value of the displacement of the tibial anatomic axis to the femoral anatomic axis, measured at the best estimate of the joint line which is 1.5 cm proximal to the fibular head); 5) FDR (anatomical axis displacement/width of the femoral condyles at their widest point); and 6) anatomical axis displacement direction (neutral, lateral or medial). The rationale for and specific steps in obtaining these measurements have been extensively discussed by Ziran et al. [11]. However, intra- and interobserver reliability of TWR and FDR have never been assessed to the best of our knowledge. To estimate it, the same method previously described for Schatzker and AO/OTA classifications reliability assessment was used.

### Statistical analysis

Clinical characteristics of patients and fractures with and without ACS were compared using frequency distributions for discrete variables and means and ranges for continuous variables. Univariate odds ratios (OR) and 95% confidence intervals (95% CI) for ACS were obtained for each clinical characteristic. For this analysis, we dichotomized continuous variables at approximate midpoints (age < 45 years, TWR ≥1.05, FDR ≥0.08).

The chi-square test for linear trend was used to evaluate ACS occurrence according to soft tissue injury classified in three categories (closed fractures, open fractures Gustilo type 1 and 2, and open fractures Gustilo type 3).

Because the risk of ACS may be related to the energy dissipated in the impact, and because men and younger patients may be more likely to sustain high-energy impacts, we adjusted the other clinical variables for age and sex, using logistic regression.

Finally, we constructed a multivariable logistic regression model, starting with the most statistically significant clinical predictors, and obtained OR and 95% CIs. Two variables remained significantly related to the occurrence of ACS (see results): risks of ACS for the four subgroups of patients defined by their status on both these variables were calculated.

Intraclass correlation coefficients (ICC two-way-random) were calculated to quantify intra- and interobserver reliability in assessing Schatzker and AO/OTA classifications, TWR and FDR. The ICC is a special case of the weighted kappa and has been considered equivalent [26]. We interpreted the results following the Landis and Koch guidelines: κ <0 = poor agreement, κ 0.0–0.20 = slight agreement, κ 0.21–0.40 = fair agreement, κ 0.41–0.60 = moderate agreement, κ 0.61–0.80 = substantial or good agreement, κ 0.81–1.00 = almost perfect agreement [27].

IBM® SPSS® Statistics version 19.0.0 software (IBM SPSS, Chicago, IL) was used for statistical analysis. Statistical significance was defined as *p* < 0.05.

## Results

All 269 fractures in 265 patients had complete epidemiological and clinical data extracted from their charts, as well as complete radiographic measurements available for analysis, except for one case where radiographs were of insufficiently good quality to measure TWR, FDR, and anatomical axis displacement direction; in this case’s available radiographs, both tibial proximal metaphysis and femoral distal metaphysis were never seen together on the same antero-posterior view, making every measurement that necessitated comparison of anatomical landmarks located on both tibia and femur impossible. However, Schatzker and AO/OTA classifications could be determined for this case, as well as the presence or absence of a non-contiguous tibial shaft or pilon fracture, or knee dislocation.

**Fig. 4:**
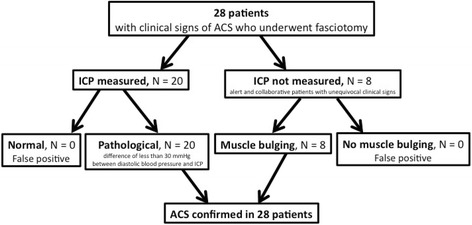
Flowchart depicting how the diagnosis of ACS was confirmed in the 28 patients with clinical signs of ACS who underwent fasciotomy. ICP monitoring was used only in patients with equivocal clinical signs and those intubated, sedated or obtunded. ACS was defined as pathological ICP values before fasciotomy, and/or by the presence of muscle bulging at the time of fasciotomy. When ICP was not measured (alert and collaborative patients with unequivocal clinical signs), muscle bulging had to be reported in the operative notes to confirm ACS. If muscle aspect was not described in the operative notes, the diagnosis of ACS was based on ICP measurements. The diagnosis of ACS was not confirmed if fasciotomy was performed without prior ICP measurements, and if there was no muscle bulging at the time of surgery. There was no false positive ACS diagnosis, meaning that every patient who underwent fasciotomy had pathological ICP values before fasciotomy, and/or presented muscle bulging at the time of fasciotomy. *ACS*: acute compartment syndrome; *ICP*: intra-compartmental pressure

Overall, ACS occurred in 28 (10.4%) of 269 tibial plateau fractures. ACS was confirmed 20 times by ICP measurements, and diagnosed eight times solely on clinical findings (muscle bulging at the time of surgery). There was no false positive ACS diagnosis, meaning that every patient who underwent fasciotomy had pathological ICP values before fasciotomy, and/or presented muscle bulging at the time of fasciotomy (Fig. 4). Four patients presented bilateral tibial plateau fractures; of them, 2 had unilateral ACS, but none had bilateral ACS. Timing of ACS occurrence is showed in Table 1. None of the patients with ACS was on therapeutic anticoagulation before the injury or during hospital stay.

**Table 1:** Timing of occurrence of acute compartment syndrome

	ACS occurence *N* = 28	Remarks
Before external fixation^a^	18	
During external fixation^a^	4	
After external fixation^a^	3	−2 cases within 24 h after surgery −1 case 10 days after plateau tibial EF and 1 day after ORIF of an associated ipsilateral pilon fracture
After definitive ORIF	3^b^	−1 single approach 2 days after injury, without transitory EF −1 double approach 7 days after injury, without transitory EF −1 double approach 18 days after injury, after transitory EF

^a^ External fixation was performed in all cases within 24 h after injury

^b^ All 3 cases occurred within 24 h after ORIF

*ACS* acute compartment syndrome, *ORIF* open reduction and internal fixation, *EF* external fixation

Table 2 shows demographics, injury characteristics and clinical and radiographic factors of the study patients. Younger age (<45 years), male sex, open fracture, non-contiguous tibia fracture or knee dislocation, higher Schatzker grade (IV-V-VI), higher AO/OTA classification (type 41-C), higher TWR (≥1.05) and higher FDR (≥0.08) were associated with an increased rate of ACS in univariate analysis (Table 3). There was a significant linear association (*p* = 0.002) between occurrence of ACS and severity of soft tissue injury: ACS occurred in 22 of 246 (8.9%) closed fractures, two of 13 (15.4%) open fractures Gustilo type 1 and 2, and four of 10 (40%) open fractures Gustilo type 3. Mechanism of injury and anatomical axis displacement direction were not significantly associated with ACS. Separate adjustment of each variable according to age and sex resulted in small changes in significance levels, except for open fracture, which lost its statistical significance (Table 3).

**Table 2:** Demographics, injury characteristics and clinical and radiographic factors of the 265 study patients with 269 tibial plateau fractures

	ACS absent, *N* = 241 (89.6%)	ACS present, *N* = 28 (10.4%)
Age (years)	49.6 ± 18.2 (16.8–92.2)	40.9 ± 15.5 (17.7–75.9)
Male sex	134 (55.6%)	23 (82.1%)
Mechanism other than fall from own height	186 (77.2%)	26 (92.9%)
Open fracture^a^	17 (7.1%)	6 (21.4%)
Associated skeletal lesion		
non-contiguous tibia fracture	1 (0.4%)	3 (10.7%)
knee dislocation	1 (0.4%)	1 (3.6%)
Schatzker classification		
type I	21 (8.7%)	2 (7.1%)
type II	76 (31.5%)	4 (14.3%)
type III	40 (16.6%)	0
type IV	35 (14.5%)	0
type V	44 (18.3%)	9 (32.1%)
type VI	25 (10.4%)	13 (46.4%)
AO/OTA classification		
41-B1	42 (17.4%)	2 (7.1%)
41-B2	41 (17.0%)	0
41-B3	89 (36.9%)	4 (14.3%)
41-C1	19 (7.9%)	3 (13.6%)
41-C2	8 (3.3%)	6 (21.4%)
41-C3	42 (17.4%)	13 (46.4%)
TWR	0.99 ± 0.06 (0.89–1.47)	1.01 ± 0.07 (0.9–1.19)
FDR	0.07 ± 0.05 (0–0.32)	0.09 ± 0.05 (0–0.2)
Medial anatomical axis displacement	19 (7.9%)	5 (17.9%)

^a^ Fractures were classified as either open or closed, without subgroup classification for the open ones

Values are expressed as mean ± SD (range) for age, TWR and FDR, and N (%) for other variables

*ACS* acute compartment syndrome, *TWR* tibial widening ratio, *FDR* femoral displacement ratio

**Table 3:** Association between variables of interest (demographics, injury characteristics and clinical and radiographic factors) and the occurrence of acute compartment syndrome

	Univariate analysis		Each variable adjusted for age and sex	
	OR (95% CI)	*P* value	OR (95% CI)	*P* value
Age < 45 years	2.93 (1.24–6.91)	0.014	-	-
Male sex	3.67 (1.35–9.98)	0.011	-	-
Mechanism other than fall from own height	3.84 (0.88–16.71)	0.072	2.55 (0.57–11.45)	0.22
Open fracture	3.59 (1.28–10.05)	0.015	2.64 (0.90–7.70)	0.076
Associated skeletal lesion	19.92 (3.47–114.44)	0.001	11.68 (1.95–69.93)	0.007
Schatzker type IV-V-VI	4.83 (1.89–12.34)	0.001	4.11 (1.58–10.66)	0.004
AO/OTA type 41-C	9.14 (3.55–23.51)	<0.001	8.08 (3.09–21.13)	<0.001
TWR ≥1.05	3.66 (1.50–8.89)	0.004	3.85 (1.52–9.74)	0.004
FDR ≥0.08	2.74 (1.22–6.15)	0.015	3.46 (1.47–8.18)	0.005
Medial axis displacement	2.66 (0.90–7.80)	0.076	2.64 (0.86–8.13)	0.090

*OR* odds ratio, *95% CI* 95% confidence interval, *Associated skeletal lesion* non-contiguous tibia fracture or knee dislocation, *TWR* tibial widening ratio, *FDR* femoral displacement ratio

In the multivariable regression model, only non-contiguous tibia fracture or knee dislocation (OR 50.0, 95% CI 6.4–391.0, *p* < 0.001) and higher AO/OTA classification (OR 12.8, 95% CI 4.3–38.7, *p* < 0.001) remained statistically significantly associated with the development of ACS. Table 4 shows the relation between both these risk factors and ACS occurrence: the risk of ACS was very high in high AO/OTA grade fractures associated with another fracture or dislocation, and very low when both risk factors were absent.

**Table 4:** Relation between variables identified by the multivariate analysis (presence of a non-contiguous tibia fracture or knee dislocation, and higher AO/OTA fracture classification) and acute compartment syndrome

	Total number of patients^a^ N	Patients with ACS^b^ N (%)
AO/OTA 41-B, no associated non-contiguous tibia fracture or knee dislocation	174	4 (2.3)
AO/OTA 41-C, no associated non-contiguous tibia fracture or knee dislocation	89	20 (22.5)
AO/OTA 41-B and associated non-contiguous tibia fracture or knee dislocation	4	2 (50.0)
AO/OTA 41-C and associated non-contiguous tibia fracture or knee dislocation	2	2 (100.0)

^a^ Number of patients, with or without ACS, presenting the variables as described in the first column

^b^ Number of patients with ACS presenting the variables as described in the first column; the percentage is calculated relative to the total number of patients presenting the same variables

*ACS* acute compartment syndrome

Intra- and interobserver reliability showed almost perfect agreement for all measurements (Table 5). Of note, one duplicate was excluded from the 30 randomly picked cases, leaving 29 cases (10.8% of 269 cases) for the final analysis. The trauma-fellowship trained orthopaedic surgeon and the orthopaedic chief resident both identified the same cases with associated knee dislocation. There were no false positive or false negative results.

**Table 5:** Intraobserver and interobserver reliability assessment of Schatzker and AO/OTA classifications, tibial widening ratio and femoral displacement ratio

	Intraobserver reliability ICC (95% CI)	Interobserver reliability ICC (95% CI)
Schatzker	0.972 (0.940–0.987)	0.917 (0.830–0.960)
AO/OTA	0.975 (0.948–0.988)	0.868 (0.738–0.936)
TWR	0.986 (0.970–0.993)	0.953 (0.902–0.978)
FDR	0.929 (0.854–0.966)	0.930 (0.857–0.967)

*ICC* intraclass correlation coefficient; values <0 represent poor agreement, values between 0.0 and 0.20 slight agreement, values between 0.21 and 0.40 fair agreement, values between 0.41 and 0.60 moderate agreement, values between 0.61 and 0.80 substantial or good agreement, and values between 0.81 and 1.00 almost perfect agreement. *95% CI* 95% confidence interval, *Schatzker* Schatzker classification, *AO/OTA* AO/OTA classification, *TWR* tibial widening ratio, *FDR* femoral displacement ratio

## Discussion

We found a risk of 10.4% for ACS occurring after tibial plateau fracture. Multivariable regression analysis individually confirmed non-contiguous tibia fracture or knee dislocation, and higher AO/OTA classification as significantly associated with ACS.

Reported incidence of ACS following tibial plateau fracture ranges from 0.7 to 12% [8–11, 28]. This incidence may even be as high as 53% in higher-energy fracture subgroups (Schatzker IV-V-VI) [8, 11–15]. This incidence seems to have increased over the past decades, possibly due to an increase in high-energy trauma as a causative injury associated with improved survival rates among severe polytrauma patients [29, 30]. In this perspective, the 212 fractures in our study (78.8%) sustained during trauma other than a fall from one’s own height, the 126 (46.8%) classified as Schatzker IV, V and VI, and the 91 (33.8%) as AO/OTA 41-C, represent a rather “modern” cohort of high-energy trauma patients and closely match the most recently reported incidence rates. Indeed, the incidence of ACS found in this study among high-energy pattern fracture types was 22 out of 126 (17.5%) Schatzker IV, V and VI fractures and 22 out of 91 (24.2%) AO/OTA 41-C fractures. Even though some ACS occurred during or after fixation (external or definitive internal fixation) and may be partly attributable to the surgery, we believe that the primary factor for developing ACS is the initial fracture with soft tissue injury, and that surgery only represents a decompensating factor. Traumatized soft tissues may indeed be vulnerable to further surgical aggression for a certain time after the initial injury. Surgical timing and protocol might therefore influence the development of ACS in the treatment of high-energy plateau tibial fractures. Common practice favors staged protocol with spanning fixators [13, 31]. A recent retrospective cohort study reporting the results of early definitive fixation (within 72 h of injury) of bicondylar tibial plateau fractures (AO/OTA 41-C) showed a favorable 2.9% incidence rate of ACS [32]. However, this study might have been biased by the fact that the choice of timing and protocol was left at the discretion of the attending surgeon, potentially leading to lower Injury Severity Score (ISS) in the early definitive fixation group (11 vs. 17, *p* = 0.024) and to a strong trend for patients with open fractures to sustain a staged protocol (28,6% open fractures in the staged protocol group vs. 5.0% in the early definitive fixation population, *p* = 0.012). Patients theoretically more at risk to develop ACS due to a higher energy trauma (higher ISS, open fracture) were also more prone to be excluded from the early total care protocol. Another retrospective study compared early external fixation (within 12 h of injury) to delayed external fixation (after 12 h) in the treatment of high-energy plateau tibial fractures [33]. Once again, the choice of timing was left to the surgeon’s discretion, and open fractures were more prone to be treated in the first 12 h than beyond (19% vs. 4% open fractures respectively, *p* = 0.045; 14% vs. 7% ACS incidence rate respectively, *p* = 0.304); this represents a potential source of bias. Thus, definitive conclusions about best practice in terms of timing and type of surgery to decrease ACS incidence rate cannot be drawn from both these studies.

All the ACS predictors highlighted by our univariate analysis are linked to the initial energy dissipated in the bone and soft tissue envelope at time of injury, possibly causing higher skeletal lesions (higher Schatzker and AO/OTA grades, higher TWR, higher FDR, non-contiguous tibia fracture or knee dislocation) and increased soft tissue envelope damage (open fracture) [4–7]. Younger age and male sex probably represent a propensity to sustain high-energy trauma, in a specific population more prone to engage in high-risk behaviour. It is noteworthy that in the past, there was a widespread wrongly reassuring theory assuming that an open fracture would relieve the pressure inside the muscle compartments and protect from the occurrence of ACS. Some reports showed that this was incorrect and that ACS could occur following an open fracture [9–11]. This parameter was even recognized as positively associated with the occurrence of ACS in tibial shaft fractures, with an incidence of ACS directly proportional to the severity of the open fracture [18, 19]. Our univariate analysis found a linear association between occurrence of ACS and severity of soft tissue injury, possibly linked with an increasing amount of fascial and muscle injury. Interestingly, none of the fractures classified as Schatzker IV evolved towards ACS in the present study. Reported incidence rates of ACS in Schatzker IV fractures vary from 0% to 53% [8, 11, 13–15]. There is possibly a subgroup of Schatzker IV fractures more prone to evolve towards ACS, but its identification is not clear and may involve specific factors that are not yet recognized.

However, this study was unable to point out an association between mechanism of injury and development of ACS, despite a trend towards statistical significance. Retrospective trauma classification into “fall from own height” or “other” (in an attempt to differentiate between low and high-energy trauma) was based on chart review and may not provide a good representation of the energy released at the time of injury. For instance, not all road traffic accidents and sports injuries are high-energy trauma, and their retrospective assessments may be inaccurate as all the details of the injury (vehicle speed, type of sports injury, etc.) may be difficult to retrieve from patients’ charts. This makes any conclusions about mechanism or energy of the injury weak.

In the multivariable regression model built for this study, non-contiguous tibia fracture or knee dislocation and higher AO/OTA classification remained independently highly associated with the development of ACS. Both these factors are hence red flags representing a high amount of energy transmitted to the injured limb, causing increased skeletal lesions (higher AO/OTA grade, non-contiguous tibia fracture or knee dislocation) and extensive soft tissue damage leading to the development of ACS.

Despite being the largest series to date to analyze the association between patient-related, fracture-related and radiological parameters in patients with tibial plateau fractures and the development of ACS, this study has several limitations: 1) its retrospective design with an attempt to differentiate between low and high-energy trauma based on chart review makes it prone to energy level misclassification, and weakens any conclusions about mechanism or energy of the injury; 2) ICP measurements were not performed on all patients presenting with tibial plateau fractures, which would have been the gold standard to diagnose or exclude ACS; this was not possible in the retrospective design of this study but could be performed in a future prospective study. This implies that there is a theoretical possibility of false negative diagnosis; however, we did not find any clinical records suspect of late ACS sequellae in the charts of patients that were not diagnosed with ACS; 3) in some occurrences, ACS was diagnosed clinically, without the use of ICP measurements, thus introducing the possibility of false positive diagnoses; however, common orthopaedic practice recognizes standard clinical examination as a valuable confirmation of ACS [20, 21]; 4) radiographs of the complete series of cases were analyzed by a single surgeon on one occasion, thus not addressing the possible issue of intra- and interobserver errors; however, intra- and interobserver reliability assessment performed on 10.8% of the cases showed almost perfect agreement for Schatzker and AO/OTA classifications, TWR and FDR; furthermore, the control of the radiographic evaluation of knee dislocation showed no false positive or false negative results; 5) Schatzker and AO/OTA classification systems may not adequately characterize some specific fracture patterns such as coronal plane fractures; new fracture classification systems analyzing the proximal tibia as a three-column or a four-quadrant structure should also be evaluated in a future study [34, 35]; and 6) as per local policy, grossly deformed limbs are aligned before radiographs are taken in order to protect vascularization of the injured limb. This implies that the radiographs may not reflect the initial deformity. However, one could expect that realigning the limb by gentle manipulation may not be sufficient to highly alter initial fracture displacement.

## Conclusions

Early recognition of injuries at risk of ACS is essential in the screening and treatment of tibial plateau fractures. Two parameters related to the occurrence of ACS in such fractures were highlighted in this study: the presence of a non-contiguous tibia fracture or knee dislocation (this parameter had not been reported yet), and higher AO/OTA classification. They may be especially useful when clinical findings are difficult to assess (doubtful clinical signs, obtunded, sedated or intubated patients), and should rise the suspicion level of the treating surgeon. In these cases, regular clinical examinations and/or ICP measurements should be performed before and after the surgery to monitor the soft tissue state even if the initial assessments were unremarkable regarding the eventual presence of ACS. However, they should not replace clinical examination that is paramount in the diagnosis of ACS in alert patients. Larger prospective studies are mandatory to confirm and refine both factors in predicting the occurrence of ACS.

## Abbreviations

95% CI: 95% confidence intervals
ACS: Acute compartment syndrome
FDR: Femoral displacement ratio
ICC: Intraclass correlation coefficient
ICP: Intra-compartmental pressure
ISS: Injury Severity Score
OR: Odds ratios (OR)
TWR: Tibial widening ratio
